# Supplementary material for: Soluble Epoxide Hydrolase Contributes to Cell Senescence and ER Stress in Aging Mice Colon
Source: Int J Mol Sci. 2023 Feb 26;24(5):4570. doi: 10.3390/ijms24054570 (PMC10003560; doi:10.3390/ijms24054570)
Supplement: Supplementary file 1 [file ijms-24-04570-s001.zip › ijms-2219938-supplementary.pdf]

Supplementary Materials

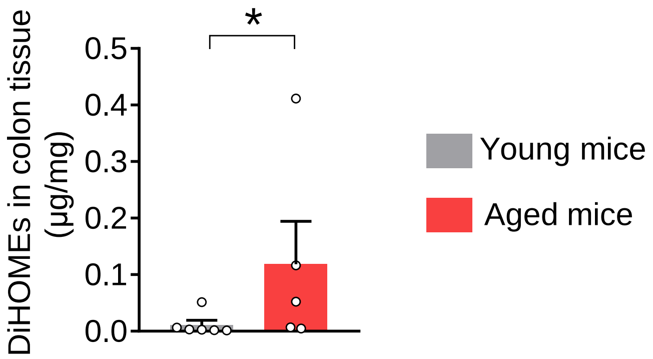

Figure S1. **The levels of DiHOMEs in the colon of young or aged mice.** The results are expressed as mean ± SEM. n = 5-6 mice per group. The statistical significance of the two groups was determined using Wilcoxon–Mann–Whitney test. * P < 0.05

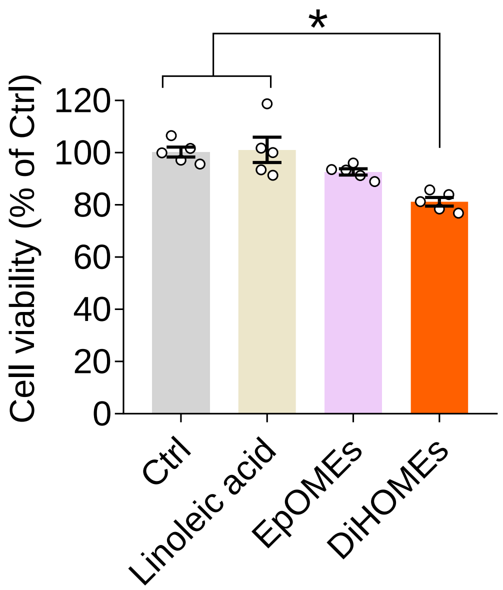

Figure S2. DiHOMEs, not linoleic acid nor EpOMEs, reduce cell viability in human colonic CCD-18co cells. The CCD-18co cells were treated with methyl esters of linoleic acid, EpOMEs, or DiHOMEs at 10 µM for 24 hours. The methyl esters were used to encourage rapid penetration of cells where the esters are rapidly hydrolyzed to the free acid anions. The results are expressed as mean ± SEM. Statistical significance of three groups was determined using one-way ANOVA. * P < 0.05.

Table S1. Sequences of primers in qRT-PCR

Gene	Forward primer	Reverse primer	Product length
**Mouse primer information**			
Gapdh	AGGTCGGTGTGAACGGATTTG (Tm = 62.6°C)	TGTAGACCATGTAGTTGAGGTCA (Tm = 62.6°C)	95 bp
Ephx2	GCGTTCGACCTTGACGGAG (Tm = 63.0°C)	TGTAGCTTTCATCCATGAGTGGT (Tm = 61.3°C)	196 bp
p21	CCTGGTGATGTCCGACCTG (Tm = 61.7°C)	CCATGAGCGCATCGCAATC (Tm = 62.0°C)	103 bp
p16	CGCAGGTTCTTGGTCACTGT (Tm = 62.4°C)	TGTTCACGAAAGCCAGAGCG (Tm = 63.0°C)	127 bp
Tp53	CTCTCCCCCGCAAAAGAAAAA (Tm = 60.8°C)	CGGAACATCTCGAAGCGTTTA (Tm = 60.4°C)	84 bp
β-galactosidase	GCACGGCATCTATAATGTCACC (Tm = 61.1°C)	GTATCGGAATGGCTGTCCATC (Tm = 60.3°C)	88 bp
Perk	AGTCCCTGCTCGAATCTTCCT (Tm = 62.4°C)	TCCCAAGGCAGAACAGATATACC (Tm = 61.2°C)	125 bp
Ire1	ACACCGACCACCGTATCTCA (Tm = 62.5°C)	TCCAACATTTGTCACTTGCTCT (Tm = 60.4°C)	110 bp
Chop	GTCCCTAGCTTGGCTGACAGA (Tm = 61.5°C)	TGGAGAGCGAGGGCTTTG (Tm = 59.3°C)	73 bp
Gadd34	GAGGGACGCCCACAACTTC (Tm = 62.6°C)	TTACCAGAGACAGGGGTAGGT (Tm = 61.5°C)	182 bp
**Human primer information**			
GAPDH	ACAACTTTGGTATCGTGGAAGG (Tm = 60.2°C)	GCCATCACGCCACAGTTTC (Tm = 61.7°C)	101 bp
CHOP	GGAAACAGAGTGGTCATTCCC (Tm = 60.3°C)	CTGCTTGAGCCGTTCATTCTC (Tm = 61.3°C)	116 bp
GADD34	ATGATGGCATGTATGGTGAGC (Tm = 60.2°C)	AACCTTGCAGTGTCCTTATCAG (Tm = 60.0°C)	120 bp
